# Conventional radiofrequency treatment in five patients with trigeminal neuralgia

**DOI:** 10.4317/medoral.17372

**Published:** 2012-12-10

**Authors:** Maite Bovaira, Miguel Peñarrocha, María Peñarrocha, Ana Calvo

**Affiliations:** 1 Anesthetist. Supervisor of the Pain Unit of the Levante Recovery and Rehabilitation Center. Valencia. Fellowship in Interventional Pain Practice; 2 Chairman of Oral Surgery, Director of the Master in Oral Surgery and Implantology. University of Valencia Medical and Dental School. Valencia; 3 Associate Professor of Oral Surgery. University of Valencia Medical and Dental School. Valencia; 4 Anesthetist. Staff physician of the Pain Unit of the Levante Recovery and Rehabilitation Center. Valencia (Spain)

## Abstract

Introduction: In trigeminal neuralgia, when drug treatment proves ineffective, other management options must be considered. In this context, conventional radiofrequency of Gasser’s ganglion is a safe and effective alternative. Material and Methods: We describe 5 patients with long-evolving trigeminal neuralgia subjected to conventional radiofrequency according to the Sweet technique, with a follow-up of two years. Results: Pain relief was complete after two months in all cases. One patient suffered an unexpected episode of nausea, vomiting and foul odor sensation that subsided after three days of rest and drug treatment. Three patients described non-painful hypoesthesia in the region of the treated nerve branch that subsided within three months. The patients remained free of symptoms over long-term follow-up. In one case the same radiofrequency technique had to be repeated after 21 months because of the reappearance of symptoms in the same zone, followed by immediate pain relief. Conclusions: In our series of patients trigeminal neuralgia was not controlled by drug treatment, and conventional radiofrequency targeted to Gasser’s ganglion proved very effective, with no major complications.

** Key words:**Trigeminal neuralgia, conventional radiofrequency, trigeminal ganglion.

## Introduction

Primary trigeminal neuralgia is one of the most painful conditions seen in human clinical practice, and while many treatments have been developed to afford pain relief, none of them are uniformly effective (1). Radiofrequency (RF) ablation is a minimally invasive technique used on an outpatient basis that has been applied to trigeminal neuralgia when drug treatment proves ineffective (2). The typical clinical characteristics of trigeminal neuralgia comprise fulgurant or stabbing pain in the form of bursts that last for seconds, with the existence of trigger factors or zones. However, not all forms of facial pain in the territory of the V cranial nerve necessarily meet all these criteria. As a result, Bruchiel established a subclassification of trigeminal neuralgia ( Table 1) (3). The most frequent form of presentation of the pain is in the territories of trigeminal branches V2 and V3 (32%), and in those cases where only one nerve branch is affected, the most common presentations correspond to V2 (17%) and V3 (15%)(1). A number of studies have compared different techniques for the treatment of trigeminal neuralgia (4). According to the most recent publications, microvascular surgery offers the best results in terms of improvement in patient quality of life and pain over the long term. The percutaneous techniques (radiofrequency, glycerol and balloon procedures) offer very good efficacy (over 90%), but with a higher relapse rate – with no major differences among them ( Table 2). In elderly patients, radiofrequency ablation is usually a better choice than surgical treatment, due to the morbidity-mortality associated with surgery (1).

**Table 1:**
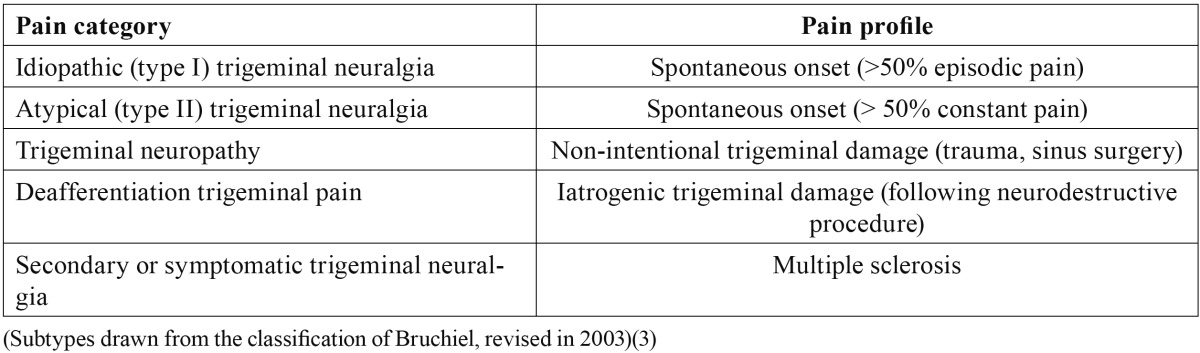
Subclassification of trigeminal neuralgia.

**Table 2:**
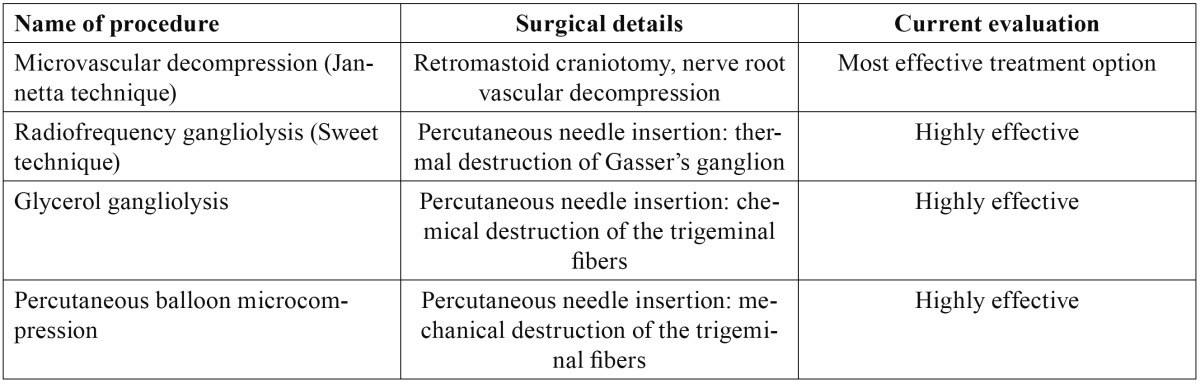
Invasive techniques for the treatment of trigeminal neuralgia.

The present study describes a series of patients with long-evolving trigeminal neuralgia subjected to conventional radiofrequency.

## Material and Methods

Between January 2006 and December 2007, we reviewed 13 patients with trigeminal neuralgia seen in a medical-surgical unit of a University clinic; of these 5, complied with the following criteria for treatment with conventional radiofrequency:

a. Typical or atypical primary trigeminal neuralgia with a duration of over two years.

b. Inefficacy of drug treatment.

c. A pain visual analog scale (VAS) score of over 7/10, with poor quality of life because of the pain.

d. Absence of concomitant disorders capable of accounting for the symptoms.

e. Normal brain magnetic resonance imaging findings.

The inclusion criteria were the obtainment of informed consent to participation in the study and a clinical follow-up of two years. The treated patients had a mean age of 64.8 years (range 55-76); there were three women and two men. The distribution according to the affected trigeminal nerve branch was as follows: four patients suffered pain in the territory of the third branch (V3)(3 right side and 1 left side), and one patient suffered pain in the region of the second branch. Two subjects had undergone extraction of a premolar in the painful quadrant before the actual onset of pain. However, in both cases the clinical manifestations of neuralgia were typical ( Table 3). Only one patient presented atypical neuralgia characterized by constant pain associated to paroxysmal episodes. In all cases the pain had been present for a long time (between 4-27 years, with a median of 11.4 years). All of the patients had received coadjuvant analgesia (carbamazepine, amitriptyline) until such treatment proved ineffective or produced side effects requiring treatment suspension ( Table 3). At the time of radiofrequency ablation, all of the patients described their pain as terrible, with a great impact upon quality of life.

**Table 3:**
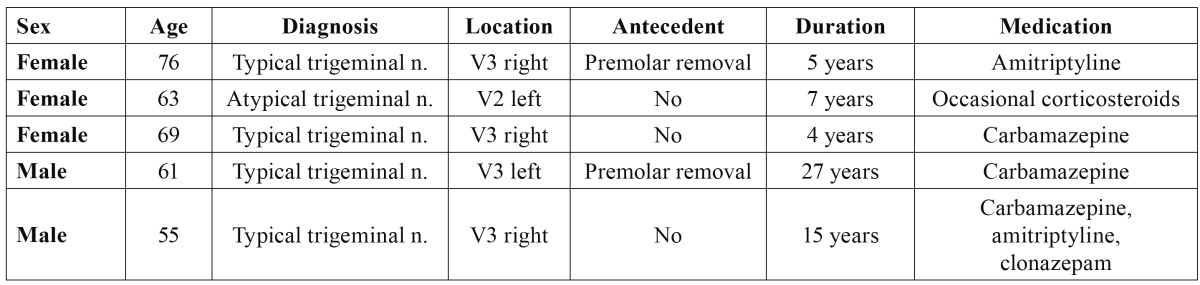
Clinical data of the study sample.

All of the patients received an explanation of the different treatment options, and accepted radiofrequency treatment of Gasser’s ganglion.

**Figure 1:**
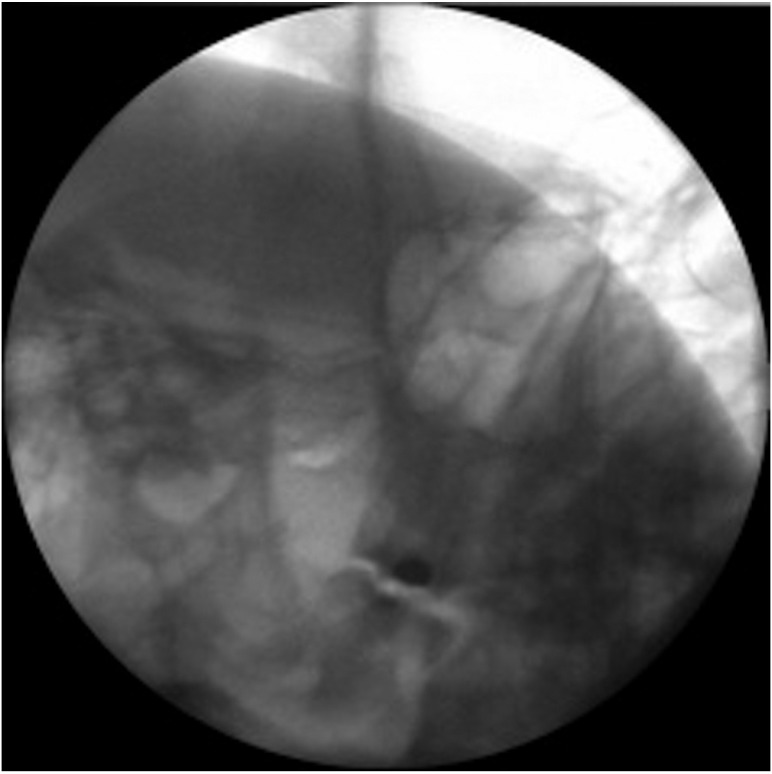
Visualization of the foramen ovale in the submental and oblique projections.

**Figure 2:**
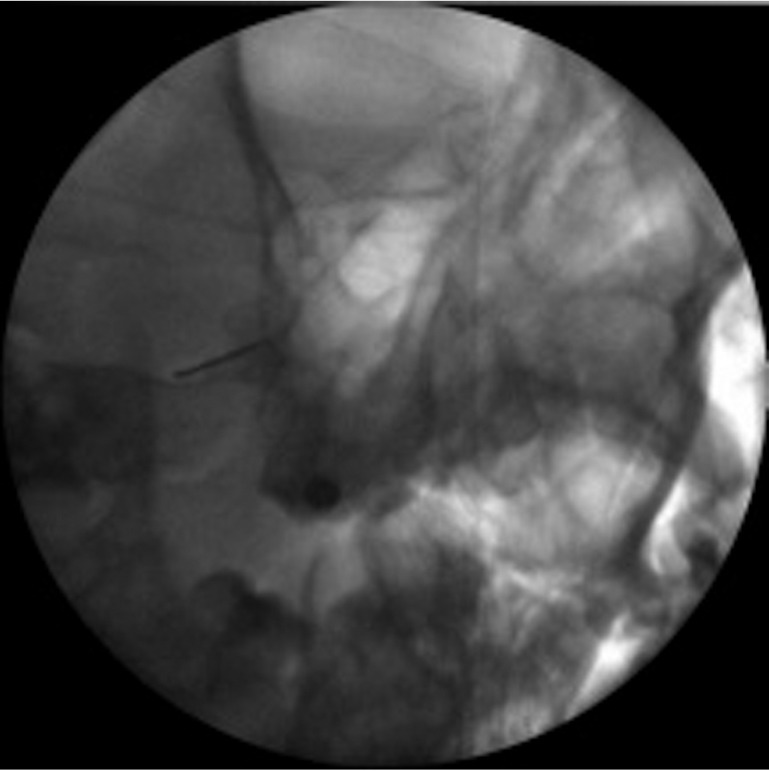
Sweet’s technique: Needle insertion through the foramen ovale, targeted to the second trigeminal branch (central).

**Figure 3:**
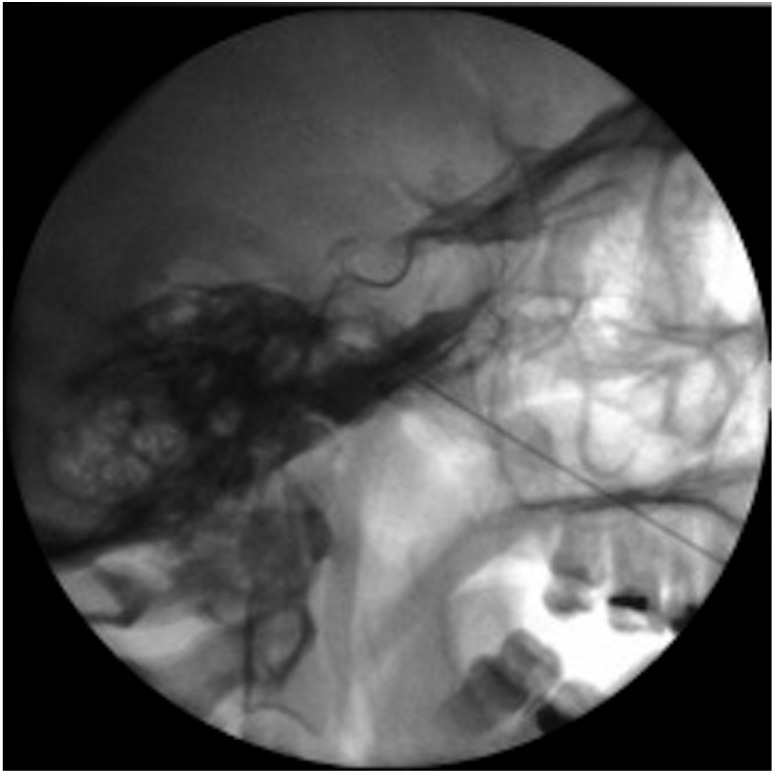
Lateral projection: confirm needle insertion depth. Observe the clivus and sella turcica.

Conventional radiofrequency targeted to Gasser’s ganglion is carried out under radioscopic control, following the technique described by Sweet (5). The patient is placed in supine decubitus, with an initial submental radioscopic projection. From here the projection is inclined obliquely towards the affected side until the foramen ovale is visualized medially with respect to the mandibular ramus and lateral to the maxilla (Fig. 1). Depending on the nerve branch requiring treatment, the target is more medial (V1 or ophthalmic) or more lateral (V3 or mandibular) (Fig. 2). A needle (Sluijter-Mehta cannula) is inserted 2-3 cm from the lateral margin of the mouth, according to whether the target is more lateral (2 cm) or more medial (3 cm). It is important to place a finger in the mouth of the patient to ensure that the needle does not perforate the oral mucosa. Following insertion, the radioscopic projection is shifted to lateral, to assess the depth to which the needle must be advanced, and to avoid moving too close to the clivus and sella turcica (Fig. 3). Sensory testing is performed to better locate the branch to be treated, with a motor test that only proves positive with the third trigeminal branch. The procedure requires deep patient sedation, and three lesions are usually made (60 seconds at 60º-70ºC), with periods of awakening and reevaluation after each of them. In the case of relapse, the procedure is repeated with the same characteristics.

## Results

The technique was carried out without problems in all patients, with positive sensitivity tests (at < 0.3 volts) and a positive motor test in the case of the third branch. Three lesions were made in the described temperature interval (60-65-75ºC). Following each lesion we checked the corneal reflex and the existence of hypoesthesia of the second and third trigeminal branches (consistent with the lesions made). The immediate postoperative course proved excellent in all cases.

Thirty hours after RF treatment, one patient suffered an unexpected episode of nausea, vomiting and foul odor sensation that subsided after three days of rest and drug treatment with ondansetron (4 mg via the oral route every 8 hours) and corticosteroids (dexamethasone 4 mg via the oral route per day). The olfactory symptoms slowly subsided, leaving persistent dull pain that dis-appeared after two months. In the rest of the patients pain relief was immediate and lasting.

Three patients described non-painful hypoesthesia in the region of the treated nerve branch that subsided within three months ( Table 4). As regards the long-term outcome, all the patients were found to be free of symptoms and without medication after 6 months. In one case the same radiofrequency technique had to be repeated after 21 months because of the reappearance of symptoms in the same zone, followed by immediate pain relief.

**Table 4:**
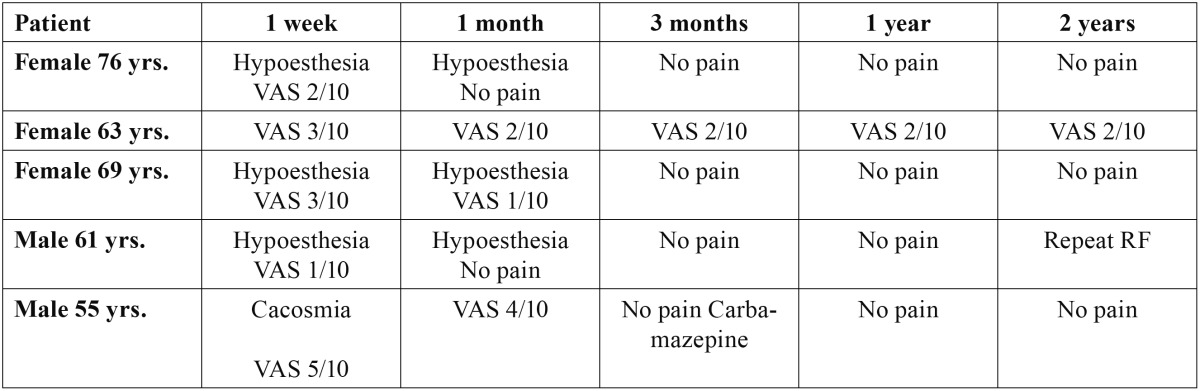
Post-treatment course.

## Discussion

Primary trigeminal neuralgia most often manifests between 50 and 70 years of age. In 90% of the cases, the symptoms first appear after age 40. The prevalence is higher in women than in men (1.5:1 proportion). In our series the women were within the established age range (4), though the males were under age 40 at the time of symptoms onset. The gender distribution was consistent with the proportion described in the literature. Typical trigeminal neuralgia must meet a series of clinical diagnostic criteria (6). In our series, four of the patients presented typical (or type I) neuralgia, while one subject suffered constant pain with associated paroxysmal episodes. When dealing with trigeminal neuralgia, we must discard other underlying disorders such as traumatisms, tumors, connective tissue diseases, infections or demyelinating disorders (7). In our patients the existence of concomitant pathology was ruled out by brain magnetic resonance imaging. Regarding distribution according to the different nerve branches, the association of V2 and V3 involvement is known to be the most common presentation, though in our series most of the subjects presented pain of the third trigeminal branch, and only one patient suffered pain corresponding to the second branch.

Regarding treatment, the initial approach always consists of drug therapy. Carbamazepine is usually able to reduce the pain by 70% (1). Other drug substances have also been used, with more irregular results, including oxcarbazepine, amitriptyline (8), gabapentin, pregabalin and baclofen. Although microvascular decompression surgery affords the best results ( Table 4), conventional radiofrequency could be an effective technique (scientific level of evidence 2B+), and would avoid the morbidity-mortality associated to surgery – particularly in elderly patients, where RF ablation therefore could be regarded as the first treatment choice (1). However, radiofrequency ablation is not without complications in the form of a diminished corneal reflex (5.7%), masseter muscle weakness or paralysis (4.1%), dysesthesia (1%), painful anesthesia (0.8%), keratitis (0,6%) or temporal paralysis of cranial nerves III and IV (0.8%).

The development of foul odor sensation following radio-frequency has not been described in the literature. We are unable to find a physiopathological explanation for this condition, recorded in one of our patients, not even when treatment is targeted to the second trigeminal branch, which conducts sensitivity from the nasal portion.

Despite the few complications of conventional radiofrequency, pulsed radiofrequency appears to be a reasonable alternative in the treatment of trigeminal neuralgia, given its non-ablative nature. However, the results obtained in the only randomized study published to date have been disappointing (9).

## Conclusion

In our series of patients trigeminal neuralgia was not controlled by drug treatment, and conventional radio-frequency targeted to Gasser’s ganglion proved very effective, with no major complications.
